# Protein Supplements and Their Relation with Nutrition, Microbiota Composition and Health: Is More Protein Always Better for Sportspeople?

**DOI:** 10.3390/nu11040829

**Published:** 2019-04-12

**Authors:** Anna Kårlund, Carlos Gómez-Gallego, Anu M. Turpeinen, Outi-Maaria Palo-oja, Hani El-Nezami, Marjukka Kolehmainen

**Affiliations:** 1 Institute of Public Health and Clinical Nutrition, University of Eastern Finland, P.O. Box 1627, FI-70211 Kuopio, Finland; carlos.gomezgallego@uef.fi (C.G.-G.); hani.el-nezami@uef.fi (H.E.-N.); marjukka.kolehmainen@uef.fi (M.K.); 2 Valio Ltd, R&D, P.O. Box 30, 00039 Valio, Finland; anu.turpeinen@valio.fi; 3 Business School, University of Eastern Finland, P.O. Box 1627, FI-70211 Kuopio, Finland; outi-maaria.palo-oja@uef.fi; 4 School of Biological Sciences, University of Hong Kong, Pok Fu Lam Road, Hong Kong SAR, China

**Keywords:** high-protein diets, amino acid, sports nutrition, gut microbiota, protein metabolism, protein fermentation, dietary supplements market

## Abstract

Sports nutrition products are developed and targeted mainly for athletes to improve their nutrient intake, performance, and muscle growth. The fastest growing consumer groups for these products are recreational sportspeople and lifestyle users. Although athletes may have elevated physiological protein requirements and they may benefit from dietary supplements, the evidence regarding the role of dietary protein and supplements in the nutrition of recreational sportspeople and sedentary populations is somewhat complex and contradictory. In high-protein diets, more undigested protein-derived constituents end up in the large intestine compared to moderate or low-protein diets, and hence, more bacterial amino acid metabolism takes place in the colon, having both positive and negative systemic and metabolic effects on the host. The aim of the present review is to summarize the impact of the high-protein products and diets on nutrition and health, in sportspeople and in sedentary consumers. We are opening the debate about the current protein intake recommendations, with an emphasis on evidence-based effects on intestinal microbiota and personalized guidelines regarding protein and amino acid supplementation in sportspeople and lifestyle consumers.

## 1. Introduction

Protein and amino acid supplements are widely marketed for athletes and habitually active consumers as muscle growth and performance-enhancing products, and high-protein, low-carbohydrate diets are traditionally applied for weight-loss purposes. However, the knowledge about the nutritional significance and effects of dietary protein and sport supplement products varies greatly among sportspeople and lifestyle users, especially in relation to individual sports activity level and overall diet and metabolic state [1,2,3]. Protein is an essential nutritional component in the human diet throughout life, as it secures growth in infancy, supports muscle and bone metabolism, ensures the maintenance and development of a normal nervous system, and helps to sustain muscle mass and physical performance in older ages, for instance. Yet, only rarely does the normal every-day Western diet not provide enough protein to meet daily requirements [4].

Athletes may have elevated physiological protein requirements, to maintain adequate protein synthesis and energy production, as well as sufficient immune function and good gut integrity in the multi-stress conditions of goal-directed, frequent, intensive and/or prolonged exercise routines. Protein need increases along with the increasing intensity and duration of an athletic performance; therefore, protein should be included in the meals before and after the actual performance and regularly during the day to secure an efficient supply of essential, or indispensable, amino acids [5]. To meet these specific nutritional requirements, several foods and supplements have been developed. For example, branched-chain amino acids (BCAA; valine, leucine, and isoleucine) supplementation is often utilized by athletes and has been proposed to reduce muscle soreness after intensive exercise and to improve training performance [6]. BCAA supplementation might have a role in regulating some brain neurotransmitter production and thus in fatigue development during exercise [6]. Furthermore, due to fast digestion and absorption, whey protein supplements are a popular protein source for athletes [7].

It is very common for regular gym goers, too, to consume protein, amino acid, and creatine supplements [1]. Often these supplements are consumed in addition to other protein-rich foods, and without any guidance from professional nutrition experts [1]. The workload of common gym attendees rarely reaches the level of professional athletes, and thus it has been suggested that the protein requirements for regular people with active lifestyles do not differ from the guidelines given for the average adult population [1]. However, recommendations are not clear and sometimes they are inconsistent; that is, in a recent review on protein requirements in elderly and obese people, it has also been proposed that the current recommendations for daily protein intake may not be adequate to support health, weight management, and healthy eating habits of aging and sedentary populations [8]. Furthermore, diets high in protein and certain amino acids have been linked with successful weight loss and with reduced risk factors of obesity and metabolic diseases [9,10,11]. In resistance-trained subjects younger than 49 years, protein supplementation has been suggested to maximize the anabolic reactions of skeletal muscle and to enhance the adaptive response to resistance training [12], and some biomarkers of high protein intake have been found to correlate with enhanced muscle function in young adults [13]. However, the intensity of the exercise sessions, the type and source of dietary protein supplements, as well as the timing of protein and supplement intake affect the efficiency of the supplements to yield beneficial effects on muscle metabolism [12]. There is also some evidence that high intake of BCAA, for example, may induce harmful effects on human metabolism in combination with high-fat diets, excess energy intake, and/or in chronic adiposity state [2]. High-protein, low-carbohydrate diets and high-fat diets may induce harmful effects on protein and amino acid metabolism and overall metabolic health of some risk populations [14,15,16,17]. Thus, the message transmitted to the common consumers about the role of dietary protein and supplementations in getting fit and managing weight and body composition is somewhat complex and contradictory, and safe and relevant utilization of protein and amino acid supplements may require a good understanding of many physiological variables.

In high-protein diets, more undigested protein-derived constituents end up in the large intestine compared to moderate or low-protein diets, and hence, more bacterial amino acid metabolism takes place in the colon [18]. The colonic fermentation of dietary amino acids may result in end-products having systemic and metabolic effects on the host, and these effects can be both positive and negative [18,19,20]. Furthermore, some weight-loss diets may also promote metabolite profiles that are likely to be detrimental to colonic health [17]. The development of biomarkers for protein fermentation could aid the assessment of optimal daily protein intake and personalized protein nourishment [21], and thus, gut microbiota deserves attention when regarding personalized nutrition.

The aim of the present review is to summarize the impact of the high-protein products and diets on nutrition and health, in sportspeople and in sedentary consumers. We are opening the debate about the current recommendations in this area with an emphasis on evidence-based effects on intestinal microbiota and personalized guidelines regarding dietary recommendations and supplementation in sportspeople and lifestyle consumers.

## 2. Consumption and Trends in Food and Supplements Intended for Sportspeople: The Market Perspective

Whilst sports nutrition products are developed and targeted mainly for athletes to improve their nutrient intake, performance, and muscle growth, the fastest growing segments for these products are recreational sportspeople and lifestyle users. This has been considered by the regulatory authorities in the European Union in the Report from the Commission to the European Parliament and the Council on Food Intended for Sportspeople [22]. The report defines ‘sportspeople’ as “people who practice sport once a week or more” and ´lifestyle users´ as people consuming food and supplements intended for sportspeople but “practice sport less than once a week or not at all”. The growth of the industry owes much to increasing health awareness, but also to consumers’ easy access to sports nutrition products, such as nutritional supplements, energy and nutritional bars, protein bars and powders, sports drinks and gels, non-carbonated and non-caffeinated sports beverages and nutritional supplements [23]. As sport nutrition is mostly focused on the improvement of athletic performance, the impact of these food supplements on health of recreational sportspeople and lifestyle users should be carefully considered.

**Figure 1:**
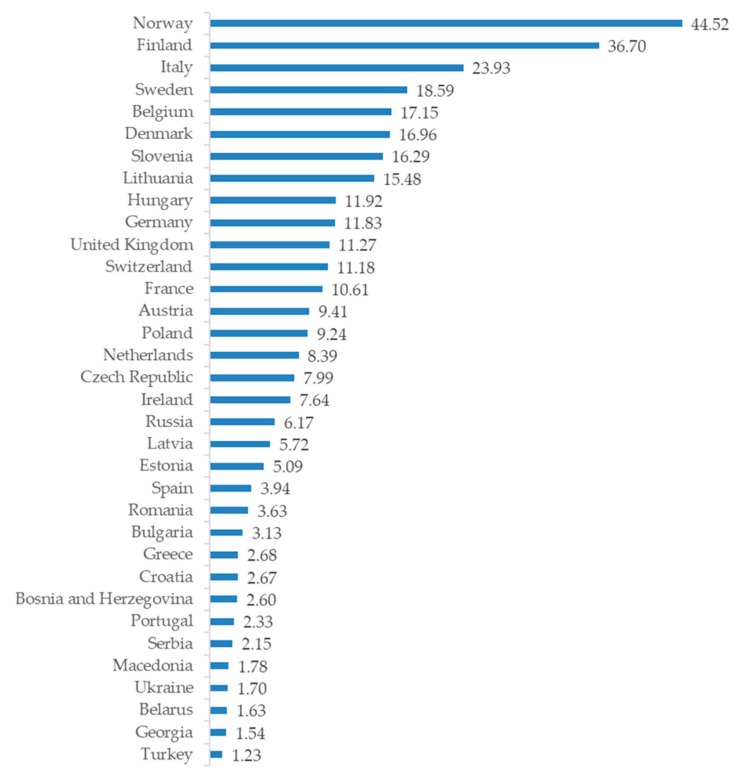
Value of the dietary supplements market per capita in Europe in 2015 (€).

In the European Union, food supplements have been defined as follows: “foodstuffs the purpose of which is to supplement the normal diet and which are concentrated sources of nutrients or other substances with a nutritional or physiological effect, alone or in combination, marketed in dose form, namely forms such as capsules, pastilles, tablets, pills and other similar forms, sachets of powder, ampoules of liquids, drop dispensing bottles, and other similar forms of liquids and powders designed to be taken in measured small unit quantities” [24]; a similar definition is employed by the US Food and Drug Administration [25]. The retail value of sports nutrition in the European Union reached €3.07 billion in 2014, the UK (€ 732m), Spain (€ 491m), Germany (€ 452m), and Italy (€ 358m) having the largest market shares [3]. In dietary supplements, Italy was the leading country with a market value of €1.4 billion in 2015, followed by Germany (€ 967m), Russia (€ 888m), and the UK (€ 737m). The fastest growth in market value is expected in Eastern European countries such as Romania, Turkey, Bosnia-Herzegovina, Russia and Macedonia [26]. Compared to the population of the countries [27], however, the highest sales per capita can be found from Norway (€ 44.52), Finland (€ 36.70), and Italy (€ 23.93) (Figure 1). In the light of these numbers, even considering that more than the 40% of athletes report using supplements [28], it is clear that the sale of supplements is no longer just explained by the use of athletes and the increase in consumption is spurred by recreational sportspeople and lifestyle consumers. Increasing health consciousness, needs for a balanced diet, and drive for weight-control, have led consumers to complement their diet with food supplements [29], and for example, the value of global protein supplements market is expected to reach €19.1 billion by 2025 [30]. Despite the fact that the growth rate has leveled off to 9.5 %, growth in the supplement industry is still fueled by the aging population, fitness trends, growing interests in plant-based protein supplements, accessibility to e-commerce, and continuous interests in self-care [31]. However, North America continues to be the highest revenue-generating region in the world and the developing countries, where health consciousness is coupled with rising disposable income, is triggering the fastest overall growth of the industry [23].

A broader basket of consumers for sports nutrition has increased the need for consumer protection and regulation [32,33]. In 2016, the European Union agreed that sports nutrition is regulated under the General Foods Law provision and no specific regulation is needed. At the international level, the regulation is fragmented and varies between markets, the European Food Safety Authority’s (EFSA) Nutrition and Health Claims Regulation (NHCR) being one of the strictest [34]. The availability of cheap and counterfeit products challenges the industry, especially in developing markets [23] but illegal ingredients such as those banned by the World Anti-Doping Agency (WADA) have been found worldwide (see e.g., references [35,36,37]). Marketing of supplements for extending beyond sportsmen has increased ethical concerns [38,39].

## 3. Protein and Amino acid Supplements Targeted for Better Athletic Performance

### 3.1. Protein Intake and General Recommendations for Healthy Adults and Athletes

In the US, the recommended dietary allowance for protein in the average population is 0.8 g protein/kg/day [40]. In the European Union, based on nitrogen balance data, EFSA has stablished the average requirement of 0.66 g protein/kg body weight per day for healthy adults regardless of sex; thus, the recommendation for daily intake has been set to 0.83 g protein/kg body weight per day [41], which is around 10%–12% of total energy intake (E%) [42]. However, these recommendations are different at the national level and, as an example, the Spanish recommendation is 0.93–1.2 g protein/kg/day [43] and the Finnish recommendation is 1.1–1.3 protein/kg/day [44]. The mean protein intake in adults among the different European countries ranges from 13.7 E% in Latvia to the 17.6–19 E% in Portugal, being above the recommendations in all the countries [41].

However, recommendations become more heterogeneous and inconsistent when the focus is on athletes and sportspeople (and other target groups involved in regular or irregular intense muscular exercise). For endurance- and strength-trained athletes, the Position of the American Dietetic Association, Dietitians of Canada, and the American College of Sports Medicine has recommended a protein intake of 1.2–1.7 g/kg/day [45]. Sophisticated nitrogen balance studies have suggested a recommended protein intake of 1.5–2.0 g protein/kg/day for strength and power athletes [46], and for endurance athletes an intake of 1.83 g protein/kg/day has been recommended in a study employing an indicator amino acid oxidation method [40]; however, in these studies, only a very limited number of subjects have been studied. Thus, depending on the recommendation, athletes may need almost twice as much protein as the more sedentary population to maintain protein synthesis, adequate energy production, and sufficient immune function and gut integrity over the exercise-induced stress [6]. In the European Union, EFSA does not consider necessary any specific recommendation for sportspeople beyond a well-balanced diet, because if the protein contribution to total energy intake is kept at about 10–12 E%, a higher intake of energy to meet the requirements of higher physical activity will come along with a higher intake of protein [42].

Protein need increases along with increasing intensity and duration of performance. Earlier recommendations focused more on total protein intake during the day, whereas optimal timing of protein intake is now also highlighted [47]. Protein should be included in meals before and after the actual performance and regularly (every 3–5 h) during the day to secure the efficient supply of essential amino acids (EAA) [47]. Although recommendations on protein intake are given separately for endurance and strength athletes, more important is to adapt the intake according to the needs of different training periods [5]. Another important issue is the adequate energy intake, to ensure that amino acids are used for protein synthesis and are not oxidized [48]. It is important to consider the fact that manipulation of dietary protein and fat intake may have a higher impact than carbohydrates in optimizing body weight and body composition in athletes, but this effect can be different according to genetic variations [49].

If the diet of sportspeople lacks protein, several effects on organ systems may take place [50]. Adequate protein intake is supposed to support bone metabolism [51] and body protein maintenance [40], for example, and these aspects further promote good athletic performance and injury prevention [52]. In extreme cases, lack of protein could also cause menstrual disorders in female athletes [53]. However, data regarding incidence of protein deficiencies in sportspeople are scarce, probably because of the lack of specific tools and biomarkers for monitoring athletes’ nutritional status [54].

There is only limited information available concerning the possible adverse effects of long-term protein supplement utilization; this highlights the need for better regulation and guidance for protein supplement availability and dosage, respectively, especially for specific risk populations, such as people at risk of kidney failure [55]. Furthermore, liver and bone metabolism may be profusely challenged because of excessive protein intake [56], and future research is needed to allow clear science-based recommendations for specific population groups. 

### 3.2. Evidence-Based Effects of Protein and Amino Acid Supplements on Athletic Performance

The human body does not store amino acids like it does fatty acids or carbohydrates [57]. This means that we have to ensure that daily intake of amino acids required for protein synthesis and other specific metabolic functions is adequate. Levels of amino acids in the blood are relatively constant [57]. Thus, if dietary protein intake is suboptimal, muscle protein breakdown is increased. Respectively, in case of excessive dietary intake, proteins are catabolized and used for energy.

Consistent evidence exists that ingesting 20–30 g total protein or 10 g EAA during or after exercise results in increased muscle protein synthesis (MPS) as well as improved nitrogen balance [5]. Higher protein doses (40 g) have not been shown to further enhance MPS [58]. Protein ingestion before exercise seems to have less influence on MPS but may still enhance muscle reconditioning depending on the type of training that takes place. Consuming both protein and carbohydrate during prolonged exercise (resistance or endurance-type exercise for several hours) has been shown to stimulate MPS during the exercise period and to result in a positive whole-body net protein balance, compared to a negative net protein balance when only ingesting carbohydrates [59]. In addition, a recent systemic review and meta-analysis has reported that the consumption of protein supplements alone or in combination with other ingredients increases fat-free mass gains after resistance exercise training [60]. However, it is important to remark that this effect was greater in untrained and elderly individuals than in trained and younger people [60].

Yet, another opportunity to enhance MPS and post-exercise recovery is protein administration before sleep. Ingestion of 30–40 g casein after evening exercise has been shown to stimulate net muscle protein accretion throughout the night and improve whole body protein balance [61] Although, MPS is most active during the first few hours post-exercise, muscle appears to be “sensitized” to protein feeding for at least 24 h after exercise [62]. 

The quality of dietary protein is also important. Athletes should consume protein with a high biological value to obtain adequate amounts of EAA [47]. Animal and especially dairy-based proteins have the highest content of EAA and greatest anabolic effect when compared to plant proteins, which typically are low in one or more EAA [63,64]. Rapidly digested proteins that contain adequate leucine (700–3000 mg) are most effective in stimulating MPS [47]. 

Whey protein, which has the highest BCAA content of natural protein sources has been shown to stimulate muscle protein synthesis in a dose-dependent manner via the mammalian target of rapamycin (mTOR) pathway [65]. Dose-dependency has been shown to plateau at approximately 2 g of leucine at rest, but to increase up to 3.5 g leucine when ingested post-exercise (see reference [47]). Increases in plasma leucine and total BCAA concentrations have been associated with improved endurance performance and upper-body power [66]. 

Accumulating data suggest that BCAA supplementation before exercise may exert positive effects of BCAA on muscle soreness and low-to-moderate exercise-induced damage in some population groups [67,68], but it remains controversial and unclear due to the small amount of studies included in systematic reviews and meta-analysis. A recent meta-analysis including eight studies with a relatively small number of participants (ranges between 12–28 subjects included in each) indicated that use of BCAAs might be better than passive recovery after various exercise types in athletes [69].

A hypothesis on central fatigue proposes that changes in the concentrations of brain neurotransmitters, specifically elevated serotonin levels and decreased dopamine levels, lead to fatigue [70]. Tyrosine is a precursor for dopamine and supplementation studies have investigated effects on performance in various types of acute stress. In their review, Jongkees et al. [71] concluded that in the few studies on tyrosine supplementation in endurance exercise, no improvements in performance were seen. Also, results from studies looking at exercise performance during heat exposure were inconsistent. Therefore, there is no consistent evidence that tyrosine improves physical exercise performance. On the other hand, tyrosine does seem to be effective at enhancing cognitive performance (such as attention) during stress and could be of benefit in sports with high cognitive demands [71]. Moderate to high intensity exercise has been shown to stimulate the hypothalamus-pituitary-adrenal (HPA) axis and to induce the release of stress and catabolic hormones [72]. Exercising above 60% maximal oxygen uptake (VO2max) induced a significant increase in circulating cortisol, while performing at low intensity (40% of VO2 max) resulted in a reduction in cortisol levels [73]. Thus, tyrosine supplementation could be most beneficial at moderate to high intensity performance level.

BCAA supplementation may also have a role in regulating brain neurotransmitter (e.g., 5–hydroxy tryptophan, dopamine, noradrenaline) production and thus in fatigue development during exercise [6]. BCAAs compete with large, neutral amino acid (LNAA) transport at the blood-brain barrier. Consequently, high serum BCAA concentrations could decrease brain LNAA uptake and thereby the synthesis and the release of neurotransmitters derived from LNAAs, notably serotonin (from tryptophan) and catecholamines (from tyrosine and phenylalanine). However, evidence that this occurs with BCAA supplements is weak [6]. Thus, currently, specific amino acid recommendations that could reduce disorders such as central fatigue syndrome cannot be given. 

It is important to remark that most of the studies about the effect of protein and amino acids supplements has been conducted in athletes or highly trained people, and the evidence for other groups involved in regular or less frequent exercise is not clear.

## 4. Gut Microbiota—Dietary Protein Interaction in Sports Nutrition

### 4.1. The Fundamentals in Microbiota—Protein Interaction

The mammalian gut microbiota is a dynamic and complex entity that consists mainly of bacteria, both symbiotic and potentially pathogenic species (500–1000 species; 10^8^ or 10^11^ in the small or large intestine, respectively) [74,75,76]. Over 90% of gut microbiota are symbiotic bacteria of which a major portion consists of anaerobes able to modulate metabolic processes of the host [77,78]. Both spatial and temporal differences exist in the diversity and number of microbial taxa in the human gastro-intestinal (GI) tract, the bacterial community being more diverse and abundant in the colon and rectal area in comparison to the esophagus, the stomach and the small intestine [79]. More than 2100 bacterial species have been isolated from human GI tract, the main bacterial phylum detected in feces being Bacteroides and Firmicutes, followed by Proteobacteria, Actinobacteria and Verrucomicrobia [80,81]. Intestinal microbial communities seem to be relatively stable over time in adults [82], but large-scale investigations of the human microbiome have revealed great variability in microbial community structure and function across different subjects [83], comprising a unique ‘fingerprint’. Factors shaping intestinal microbiota are related with host genetics and epigenetics, ethnic origin, age, gender, antibiotics exposure, history of previous disease, dietary exposures and lifestyle [84]. This high variability and diversity contrasts with the high functional redundancy mainly shaped by diet and lifestyle among other factors [81]. 

The host, the gut microbiota, and several diet-related components form a network in which microbial metabolites serve as signaling agents. In fact, gut microbiota can be referred to as a metabolic organ of high importance [78]. The gut microbiota participates in the regulation of host physiological processes and has a central role in the maintenance of intestinal homeostasis, nutrient absorption, and synthesis of EAA and vitamins [85]. Recent publications suggest that intestinal microbiota respond differently to a dietary intervention according to their composition, thereby conditioning host response [84]. The assessment of bacterial composition and function may help to predict the responses to specific dietary intervention, and open the door to personalized nutrition, based on intestinal microbiota. Recent studies have demonstrated the possibility to predict glycemic response to food consumption based on microbiota composition [86,87], but we are still far from making efficient personalized nutritional recommendations according to gut microbiota profiles. Better understanding of the factors implicating the interindividual variability in gut microbiota and their response to diet is still needed, but eventually, it will help to improve the comprehension of the impact of dietary interventions on health.

The main source of nutrients arriving to colonic microbiota is composed by a large diversity of complex glycans including undigestible polysaccharides (cellulose, hemicellulose, lignin, resistant starch pectin and oligosaccharides) but also monosaccharides and disaccharides not fully absorbed in the upper part of the GI tract and some endogenous substrates such as mucins and mucopolysaccharides [88]. In addition, some of the anaerobic colonic bacteria can use amino acid fermentation to produce energy strictly or in combination with carbohydrate fermentation [89]. For this reason, the amino acids derived from food or released from endogenous sources and arriving at the colon can serve as an amino acid source for protein fermenters [21]. *Bacteroides*, *Prevotella*, *Ruminococcus*, *Roseburia*, *Faecalibacterium* and *Bifidobacterium* are the main genera fermenting dietary fiber and vegetable oligosaccharides; *Lactobacillus* and *Bifidobacterium* can ferment lactose and human milk oligosaccharides; and *Akkermansia* and *Bacteroides* ferment mucins and mucopolysaccharides [88]. On the other hand, the main protein fermenters in the colon are species from the genera *Clostridium*, *Desulfovibrio*, *Peptostreptococcus*, *Acidaminococcus*, *Veillonella*, *Propionibacterium*, *Bacillus*, *Bacteroides* and *Staphylococcus* [21], and others belonging to the Proteobacteria phylum [90]. The bacterial fermentation of dietary amino acids may result in end-products, such as ammonia, biogenic amines and indolic and phenolic compounds among others, having systemic and metabolic effects in the host [18], and possibly impacting immunomodulatory, neurological, cardiovascular, and gut functions [18,20,91]. These metabolic products may increase inflammatory response and tissue permeability and might be implicated in the development and severity of the symptoms of colorectal cancer and metabolic diseases, including obesity, diabetes, and non-alcoholic fatty liver disease [90].

**Figure 2:**
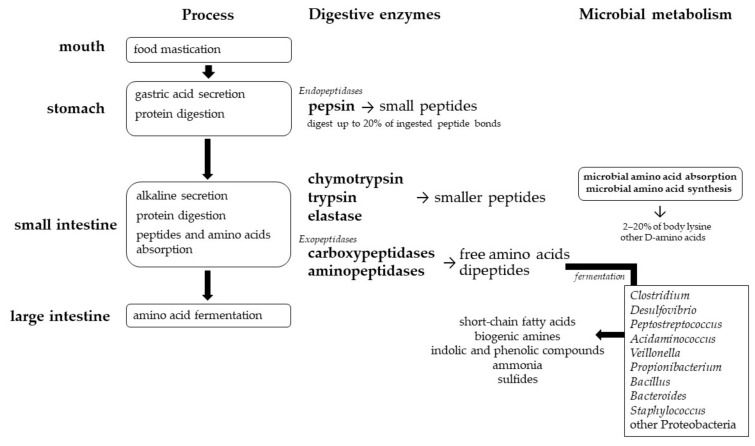
Physiological and microbial protein digestion, amino acid production and synthesis along the gastrointestinal tract.

In addition, microbial fermentation of several amino acids in the large intestine has been proved to contribute to the precursor pool of short-chain fatty acids (SCFA) [85,92]. The most abundant SCFA in the colon are derived from carbohydrates, and they are acetate, butyrate and propionate [93]. All of them have well-reported health effects and microbiota-derived butyrate, for example, regulates the energy metabolism of colonocytes by acting as an energy source [94], while propionate participates in the hormonal downregulation of energy intake [95]. On the other hand, amino acid fermentation produces branched-chain fatty acids (valerate, isovalerate, isobutyrate and 2-methyl butyrate) [74,93]. They are exclusively produced through microbial fermentation and, for this reason, can be consider reliable biomarkers of proteolytic fermentation [90]. In contrast to the extensively studied role of carbohydrate-derived SCFA, the effects of amino acid-derived SCFA on host physiology are not well known [92], and are associated with the production of other potentially harmful metabolites including ammonia, sulfides and biogenic amines, among others [21]. The physiological and microbial processes related to dietary protein metabolism in the human GI tract are presented in Figure 2.

Proteolytic fermentation in the colon depends on ecological and dietary factors [90]. Among the main factors that will condition the amount of proteins arriving to the colon, the total amount of protein ingested and the protein digestibility, bioavailability and absorption in the small intestine, may have significant importance. The dietary source and processing affect protein absorption rate [96], and in general, proteins from plant sources are less digestible in the human GI system in comparison to animal-derived proteins [97]. Especially in mixed diets high in protein, plant proteins are more readily available for fermentation in the more distal parts of the gut: these proteins are often incompletely digested in the small intestine while more digestible proteins are used to meet systemic nitrogen requirements [78,97]. However, it has been suggested that it is primarily the amount of dietary protein rather than the protein source that has a greater effect (1) on the amount of protein entering the colon, (2) on the extent of protein fermentation, and (3) on the composition of gut microbiota; this should be carefully considered in high-protein diets. 

Moreover, in vitro experiments suggest different bacterial preference between peptide fermentation and amino acids fermentation, and not all amino acids are equally suitable for fermentation [90]. In high-protein diets, overall more undigested protein-derived constituents end up in the large intestine in relation to moderate or low-protein diets [18]. As a consequence, the amounts of protein-fermenting bacteria and their metabolites tend to further increase, while the abundance of carbohydrate-fermenting bacteria tends to decrease [98,99]. In addition, dietary fiber impacts protein fermentation in the colon and can modulate relative abundance of microbial species responsible for proteolytic fermentation, attenuating the effect of the high-protein intake [90]. Because carbohydrates are the preferred carbon source for many gut microbes, protein fermentation might be invoked mainly if no fermentable fiber is available [100]. According to this, high-fiber intake can be recommended in a high-protein diet to reduce the protein impact of colon microbiota, but further studies are needed, mainly on long-term protein consumption.

The alterations in the composition and metabolism of the gastro-intestinal (GI) microbiota responsible for amino acid fermentation may indicate changes in diet and further lead to changes in host metabolism and energy homeostasis. A small study evaluating the impact of high-protein supplements on athletes’ microbiota (*N* = 24) during 10 weeks did not find any changes in microbial diversity or amino acid fermentation-derived metabolites, but a reduction in some bacterial groups including *Roseburia*, *Blautia*, and *Bifidobacterium longum* [101]. In contrast, another study shows a positive association among microbial diversity with protein intake and exercise performance [7]. However, the association between microbiota diversity and health status is not clear [102], and these relatively small datasets are not enough to make any general conclusion, although they indicate a reduction in some microbial species and an increase in overall microbial diversity. Nevertheless, the study performed by Moreno-Perez et al. [101] suggests that long-term protein supplementation may have a negative impact on gut microbiota of athletes and, consequently, have negative repercussions on athlete’s future health. Moreover, to our knowledge, there are no studies evaluating the impact of long-term high-protein supplement consumption on intestinal microbiota and amino acid fermentation in recreational sportspeople and lifestyle users of protein supplements, and due to the increased market and consumptions, this should be evaluated.

In addition to the differences in protein digestibility due to protein source, processing factors, or oversupply of dietary protein, the variable capacities of people to lyse proteins may affect the abundance in which intact or partially degraded proteins are transferred to the large intestine [21] highlighting the need for personalized nutritional recommendations. A person’s ability to digest proteins depends on their age, state of health, and on their so-called digestive phenotype, for instance [21]: protein absorption and metabolism are strongly regulated by anatomical differences, and by the fluctuations in the physiology and biochemical environments of the GI tracts of individuals [103]. From the point of view of personalized nutrition, potential indicators of excessive protein metabolism in the gut, for example, frequent and severe abdominal pain [104], abdominal fullness, bloating and feeling of distension [105], defecation urgency [106], and the production of foul-smelling flatus [107], should be taken into account, and the dietary interventions should be adjusted accordingly.

### 4.2. Microbiota Contributes to the Protein Nutrition and Gut Well-Being of Athletes

There seems to be a bit of a paradox regarding the high-protein diets of athletes. Although the physiological dietary protein requirements are elevated for athletes, high-protein diets may affect the gut microbiota and protein fermentation levels [6], with consequences for health. As a result of voluminous protein intake, more peptides enter the systemic circulation and the distal parts of the GI tract, and proteolytic fermentation may occur; this may emphasize GI-related symptoms, cause immune system disturbance and promote inflammation, damage, and dysfunction in the GI tract [108]. A tryptophan metabolite nicotinamide participates in gut microbiota regulation by activating mTOR and thus downregulating the synthesis of antimicrobial peptides [50,109]. Although activation of mTOR may facilitate muscle growth [47] and inhibition of inflammatory responses [50], over-activation may lead to gut microbial dysbiosis because of down-regulated production of antimicrobial peptides, and further, to an increase in gut permeability [110]. 

Moreover, as the nitrogen levels of the large intestinal lumen increase during intensive exercise because of protein catabolism and sports-induced stress, more nitrogen is available for gut microbiota metabolism and proliferation, further contributing to the possible shifts in microbiota composition [6]. However, in the guts of elite athletes, microbial diversity has been also found to positively correlate with protein intake and exercise [7], and thus, exercise training is suggested to help maintain a healthy and balanced gut microbiota, and muscle mass and function, which also applies for the general population [111]. It must be remembered, however, that elite athletes represent a special population, and many of them receive dietary consultation from professional nutritionists and physicians. 

In personalized nutrition plans for athletes, the effects of diet on the gut microbiota should be taken into account [6], in addition to the use of specific probiotic and prebiotic formulations. Microbiota contributes to amino acid absorption and synthesis and participates in energetic pathway regulation in skeletal muscle which may affect, for instance, muscle metabolism, size and composition [111,112]. Microbially produced amino acids, mainly absorbed in the small intestine, may be significant contributors to the plasma amino acid pool [113]. Moreover, microbial metabolites of phenylalanine and tryptophan, such as cinnamic acid, hydrocinnamate, and indolepropionate, have been associated with both alterations in the gut microbiota and reduction in muscle quality (leg press one repetition maximum/total lean mass) of healthy, young adults (age 18–35, body mass index (BMI) 19–31 kg·m^−2^) [13], and thus, should be taken into account in nutritional assessment for athletes and sportspeople. Supplementation with both protein and probiotic *Bacillus coagulans* GBI-30, 6086 has been found to decrease recovery time after 24 and 72 h in a strength exercise study protocol, and to reduce muscle damage, muscle soreness, and decline in peak power [114]. The negative effects of high-protein diets and harmful amino acid fermentation products might also be reduced by adding prebiotics, such as resistant starch, vegetables, and fiber, to the diet [6] as this promotes gut colonization by benign microbes, increases saccharolytic fermentation, decreases protein fermentation, increases GI inflammation control, and gut permeability reduction [108]. It has been suggested that aiming at an increase in the microbiota diversity and the proportion of Actinobacteria before severe, prolonged exercise should benefit gut well-being, while the amount of Proteobacteria should be a target of reduction [115]. According to these observations, if training-related gut discomfort occurs, the diet and dietary supplementations should be modified to better support a balanced metabolism and composition of gut microbiota and yet providing enough protein and EAA for muscle metabolism. Well-balanced protein intake should support both the well-being of the gut and an optimized development in athletic performance. 

## 5. The Risks of Unsupervised Protein and Amino Acid Supplements on Lifestyle Consumers

Among protein supplements, those containing BCAA are rising in popularity among consumers and have attracted the attention of the sports nutrition industry. Circulating BCAA have been related to the improvements in muscle function of young adults [13], and furthermore, diets high in protein and BCAA have been linked with successful weight loss and reduced risk to develop obesity, respectively [9,11]. Dietary supplementation with several amino acids (incl. BCAA, aromatic amino acids, lysine, threonine, cysteine, and methionine) has also been found to strongly decrease the fasting and postprandial blood glucose levels in normal-weight elderly subjects diagnosed with type 2 diabetes [10]. The amino acid supplementation may have beneficial effects on the satiation signaling and energy intake [9], and the muscle metabolism supporting blood glucose control [10]. However, the impact of oral supplementation of BCAA in the modulation of metabolic risk seems to be influenced by the dietary environment exposure [116]. The potential health benefits have contributed to the popularity of these products. 

Nevertheless, high plasma levels of BCAA have been associated with the development of type 2 diabetes and non-alcoholic fatty liver disease [117,118]. There are also indications that some high-protein low-carbohydrate diets and high-fat diets may induce detrimental effects on both gut microbiota and host protein and amino acid metabolism and further, may disturb the metabolic health of some risk populations [14,15,16,17]. Moreover, higher levels of some microbial metabolites produced after protein fermentation, such as ammonia, p-cresol or indol, have been associated with cancer development and psychiatric diseases [90]. 

Obesity, diabetes, and inflammation are often related to each other, as well as to microbiota dysbiosis [109]. Gut microbiota dysbiosis may, for instance, alter the host energy homeostasis and facilitate fat accumulation in adipose tissue by interfering with insulin sensitivity [119]. A chronic activation of mTOR in human subjects consuming a high-fat diet with BCAA supplementation has been found, and this activation is suggested to promote the development of insulin resistance [16]. Thus, it could be proposed that the overactivation of mTOR in these situations could promote dysbiosis and partially explain the inflammatory reactions often related to diabetes. A clear association between plasma BCAA concentration and incidence of type 2 diabetes has been detected in a large multiethnic population, although the strength of this association varied between different ethnic groups [15]. It has been shown that ethnic background is an important factor affecting the gut microbiota of individuals living even in the same geographical area [120], and that the ethnic origin-specific gut microbiota composition and diversity has a role in diabetes prevalence, for example [121]. It has also been reported that the gut microbiota of high BMI individuals differs from the gut microbiota of elite athletes [7], and the protein supplements may lead to the production of different metabolites with different health consequences. The gut microbiota of individuals living in the urbanizing populations of developing countries often start to resemble those of people living in developed countries after consuming higher amounts of animal protein, fat, and sugar [122]. Moreover, it has been established that migration from rural communities to urban environments causes loss in gut microbiota diversity and fiber degradation capacity and exposes the immigrants to obesity and metabolic diseases [123]. 

Thus, it can be proposed that the gut microbiota composition, versatility, and metabolism at least partially explain the higher risk of metabolic diseases in specific groups, and that in the loose frame set by the individual’s ethnic background, the microbiota and its metabolism are modified to correspond to energy and nutrient availability, and to the host’s body composition and exercise routines. Of course, it must be also remembered that the individual physiological differences may further affect the response of the gut microbiota to protein supplements and dietary interventions and thus, the metabolic responses of the host in the host-microbiota interaction.

## 6. Conclusions

There is a heterogenicity among health authorities in their recommendations on protein intake for sedentary people, sportspeople and athletes. For example, the national recommendations within the EU or those provided by nutritionist and medical associations may vary considerably and exceed the recommendations given by EFSA. The scientific community widely supports moderate intake of proteins, although some researchers have found that the current recommendations may be inadequate for athletes, elderly people, or in the case of weight-control and weight reduction diets.

High-protein diets may temporarily help to reduce weight, but there is a lack of knowledge about the long-term effects of high protein intake. In addition, the variability in the requirements and physiological impacts of high-protein diets may be an object of personalized recommendations in the near future. In any case, when a high-protein diet is recommended, special attention should be paid to the origin of these proteins and the overall quality of the food. The consumption of ultraprocessed foods has been associated with the higher prevalence of several diseases, possibly due to high content of processed vegetable fats, sugars, salt and artificial sweeteners among other components. When it comes to protein and amino acid supplements, these other components present in these products may induce adverse effects during long-term consumption, and lifestyle and recreational sportspeople might be more susceptible than athletes. We suggest that instead of adding protein and amino acid supplements to high-protein diets, protein should be preferably received from whole foods, such as fish, eggs, dairy products, legumes, and cereals, along with fibers and other food components supporting the well-being of both the host and their gut microbiota. This should be highlighted in the nutritional plans of athletes, sportspeople, as well as more sedentary populations. In addition, the marketing and advertising of high-protein and amino acid products should be carefully planned and directed according to protein needs. In our opinion, supplements should be an occasional resource to improve athletic performance and recovery in the cases supported by scientific evidence and when these benefits are difficult to achieve with regular foods. 

Considering the complex interactions within protein digestion and absorption with the host and microbial community, more information is needed to personalize the nutritional recommendations for athletes, sportspeople and lifestyle consumers regarding high-protein supplement consumption. Nutritional recommendations should better consider the variable requirements of protein intake, and personalized protein nutrition needs to be regarded in the light of genetic background, diet, lifestyle, and microbiota of the individual. The current recommendations supply an adequate amount of protein for most people, regardless of their activity level, but in specific cases there might be a need for higher protein intake. However, for many people, a protein intake higher that the current recommendations will not provide any additional benefit, and for some, it might have a negative impact on health.

### 6.1. Key Points

- Due to increasing health awareness and consumers’ easy access, the fastest-growing consumer groups for sport supplement products are recreational and lifestyle users.
- Over-consumption of dietary protein may have harmful effects on human metabolism and gut comfort, especially in combination with otherwise unbalanced or restrictive diets.
- The gut microbiota and its metabolism vary according to ethnic background, age, diet, exercise routines, geographical habitat, and individual physiological features of the host, and affect the individual’s metabolic response to dietary protein and amino acid intake.
- While studies on athletes have shown that protein and amino acid supplements may increase MPS and reduce fatigue, muscle soreness and low-to-moderate exercise-induced damage, current studies showing clear negative effects associated with high-protein diets or, e.g. BCAA supplements, are mainly reported in subjects with some type of metabolic disturbances.
- In addition to protein quality and quantity, people must pay attention to other components of their diet and maintain normal weight and physical activity to ensure the supply of essential amino acids while indulging their versatile microflora and limiting the production of potentially harmful fermentation products.

### 6.2. Future Considerations

- The scientific evidence on adequate protein intake for athletes and the general population should be carefully estimated and analyzed.
- The availability and consumption patterns of protein and amino acid supplements should be more carefully considered in dietary guidelines, and adequate guidance on the use of these products should be provided to ensure safe and relevant utilization.
- There is a need in the field of sports nutrition for more research in microbiota modulation to maintain a healthy and versatile intestinal microbiota, to ease gut discomfort, and to enhance protein utilization pre- and post-workout and during the athletic performance.
- Personalized intervention in athletes, considering their own particularities regarding protein needs and metabolization, and microbiota composition and activity, may provide better performance and recovery.
- Identification of specific metabolic and microbiota biomarkers to predict the physiological response of the host to protein intake is needed to better enable personalized protein nutrition.
